# Supplementary material for: Health service responses and help-seeking for women experiencing violence during outbreaks in low- and middle-income settings: A scoping review
Source: PLOS Glob Public Health. 2026 Apr 30;6(4):e0004482. doi: 10.1371/journal.pgph.0004482 (PMC13132175; doi:10.1371/journal.pgph.0004482)
Supplement: S1 Table — (DOCX) [file pgph.0004482.s001.docx]

*Table 3: search strategy Medline*

| 1. **Health service search terms** | (Health service* or health system or health care or healthcare or community health or treatment or therapy or care or mental health or psychotherap* or psychological or psychosocial or reproductive health or "sexual and reproductive" or contraceptiv* or family planning or abortion or post exposure prophylaxis or PEP or support group or hospital* or humanitarian) or "delivery of health care" |
| --- | --- |
|  | AND |
| 1. **VAW search terms** | (Gender-based violence or sexual violence or GBV or SGBV or intimate partner violence or rape or "sexual abuse and exploitation" or Domestic violence or family violence or violence against women or VAW or sexual abuse) or Battered Women or Spouse Abuse |
|  | AND |
| 1. **Outbreak search terms** | (Outbrea* or epidemi* or pandemic* or public health emergency or Ebola or Filovirus or COVID-19 or Coronavirus or COVID or Zika*) or Disease Outbreaks or Hemorrhagic Fever or Zika Virus Infection/ |
|  | AND |
| 1. **LMIC search terms** | Developing Countries or developing or less* developed or under developed or underdeveloped or middle income or low* income or  underserved or under served or deprived or poor* countr* or nation? or population? or world or low* gdp or gnp or gross domestic or gross national or low middle countr* or lmic or lmics or third world or lami countr* or transitional countr* or global south or (list of all countries from LMIC expert search Medline) |
