# Supplementary material for: Health service responses and help-seeking for women experiencing violence during outbreaks in low- and middle-income settings: A scoping review
Source: PLOS Glob Public Health. 2026 Apr 30;6(4):e0004482. doi: 10.1371/journal.pgph.0004482 (PMC13132175; doi:10.1371/journal.pgph.0004482)
Supplement: S4 Table — (DOCX) [file pgph.0004482.s004.docx]

*Table 6: search strategy* ***Global Index Medicus***

| 1. **Health service search terms** | "Health service*" OR "health service* delivery" OR "health system" OR "health care" OR healthcare OR "community health" OR treatment OR therapy OR care OR "mental health" OR psychotherap* OR psychological OR psychosocial OR "reproductive health" OR "sexual and reproductive" OR contraceptiv* OR "family planning" OR abortion OR "post exposure prophylaxis" OR PEP OR "support group" OR hospital* OR humanitarian or MESH terms: "Delivery of Health Care" OR "health services" |
| --- | --- |
|  | AND |
| 1. **VAW search terms** | "Gender-based violence" OR "gender based violence" OR "sexual violence" OR GBV OR SGBV OR "sexual and gender based violence" OR "intimate partner violence" OR rape OR "sexual abuse and exploitation" OR "Domestic violence" OR "family violence" OR "violence against women" OR VAW OR "violence against women and girls" OR "sexual abuse" or MESH terms: "domestic violence" OR "battered woman" OR "partner violence" |
|  | AND |
| 1. **Outbreak search terms** | Outbrea* OR epidemi* OR pandemic* OR "public health emergency" OR Ebola OR "Ebola virus disease" OR Filovirus OR COVID-19 OR Coronavirus OR COVID OR Zika* OR MESH terms: epidemic OR Ebola OR "hemorrhagic fever" OR pandemic OR "coronavirus disease" OR "Zika fever" |
|  | AND |
| 1. **LMIC search terms** | N/A (database only contains LMIC research) |
