# Supplementary material for: Health service responses and help-seeking for women experiencing violence during outbreaks in low- and middle-income settings: A scoping review
Source: PLOS Glob Public Health. 2026 Apr 30;6(4):e0004482. doi: 10.1371/journal.pgph.0004482 (PMC13132175; doi:10.1371/journal.pgph.0004482)
Supplement: Attachment — Submitted filename: Reviewer comments_VAW_Manuscript pgph-d-25-00671.pdf [file pgph.0004482.s006.pdf]

Overall assessment:
This study shines a light on a reportedly underexplored aspect of the literature the impacts of disease outbreaks on the impact the provision of and access to health services for violence against women. The authors provide a much-needed description on the published primary studies addressing this topic in the English language, succinctly highlighting the major takeaways from the studies included. The paper includes a very helpful breakdown of the key themes in the findings reported by authors in a number of settings in LMICs and serves to highlight the considerations for future research, interventions, and advocacy. Some minor changes are needed to improve the manuscript and prevent any misinterpretations of the findings reported.

Harmonization of the language surrounding the forms of sexual violence is warranted. One suggestion would be to define all the acronyms describing the various forms of violence (GBV, VAW, SGBV) in one spot – briefly discussing the ambiguity and overlap between those concepts.

General comments
I would caution the authors against slipping from language reporting the findings of the scoping review to statements reporting on the success of certain interventions. Given that no assessment of the quality of the studies was conducted, authors should beware of attributing success to the interventions described. In the results section, the authors write on line 127-128 “the approach was successful in serving some harder to reach women”. Such statements should be reported carefully.
For example, In the results section, lines 5-6: this scoping review isn’t so much reporting women’s help-seeking behavior during the pandemic, as it is highlighting **what the literature on VAW reports** when it comes to women’s help-seeking behavior. This is an important distinction that should be made clear throughout the text.
Another example is clear on line 223: “We will first describe the prevalence of help-seeking from community members…”. This sentence reads as if the authors take it for granted that the studies cited accurately reflect that prevalence of help-seeking behavior, even though no assessment of the quality of the study and the validity of their conclusions can be made. Statements like “The authors of X study reported that…” can reduce that ambiguity.

Specific comments:
Abstract:

- The authors jump directly into the description of the scoping review objectives (line 15) without clearly stating how VAW are linked to disease outbreaks. One sentence after “Services may be disrupted or deprioritized, or woman may avoid clinical settings” is needed to lead into the scoping review objectives.

Introduction

- Line 112: The authors mention that 34 articles met the inclusion criteria and were included in the final synthesis. Yet subsequent text and figures only mention 32 articles.

Results:

- Line 27: Consider rephrasing "'Safe spaces' were also closed in non-humanitarian [*settings?*] during the pandemic…"
- Line 57: Suggested change 'There was also **one** account of VAW interventions delivered successfully despite the pandemic…."
- Line 59: Change to "locally-**led** solutions"
- Lines 59-60: Clearly specify that the 642% monthly increase is *reported* by the authors of the Kenya study. As no quality assessment was performed on the studies, one cannot take the reported improvements at face value with no discussion of the quality of these findings.
- Line 164-165: Consider clarifying "In Ethiopia, services, for example from women's affairs and health facilities, were particularly limited at the local administrative (Kebel) level."
- Line 202: Clarify the statement: "In some cases, such as cross-sectional studies, it was unclear whether the pandemic saw changes in help-seeking given that in many setting survivors rarely seek help from formal services."
