# Supplementary material for: Health service responses and help-seeking for women experiencing violence during outbreaks in low- and middle-income settings: A scoping review
Source: PLOS Glob Public Health. 2026 Apr 30;6(4):e0004482. doi: 10.1371/journal.pgph.0004482 (PMC13132175; doi:10.1371/journal.pgph.0004482)
Supplement: Attachment — Submitted filename: review_eg.docx [file pgph.0004482.s007.docx]

This scoping review aims to identify opportunities for strengthening violence against women (VAW) health services during outbreaks in low- and middle-income countries (LMICs), using a thematic synthesis approach. The objectives are clearly stated and the search strategy is well constructed. I appreciated the authors’ transparency regarding the limitations of both their review process and the included studies. However, I have significant concerns about key aspects of the methodology and the overall contribution of the article. In particular, the analytical and synthesis processes are not described in sufficient detail to assess the validity or credibility of the findings. Although the authors refer to a “thematic synthesis approach”, the themes presented appear more categorical in nature, raising questions about the depth of the analysis. Without clarity on how themes were developed, the results are not reproducible, and the review lacks the transparency expected in rigorous scoping reviews. Additionally, the discussion remains quite superficial and does not fully address the article’s stated aim, limiting the paper’s added value to the literature. Despite these major concerns, I have provided detailed comments, both major and minor, because I believe the manuscript has potential. With substantial revisions in three key areas (1. Clarifying the analytical and synthesis methodology, 2. Aligning the presentation of results with the stated synthesis approach, and 3. Significantly deepening the discussion in line with the paper’s stated aim), I encourage the authors to consider resubmitting a new version at a later stage.

**MAJOR COMMENTS**

**INTRODUCTION**

While the authors define VAW and cite statistics on IPV, it remains unclear whether the review focuses solely on IPV or considers other forms of VAW (e.g., sexual violence, femicide, trafficking, early/forced marriage, etc.) Given the broad use of the term VAW, it would be helpful to clearly define what types of violence are included within the scope of this review (and later on, in the included articles) and justify this focus. This clarification would improve the conceptual clarity and ensure alignment between the stated objectives and included evidence.

**METHODOLOGY**

**Study Design**

**Line 73**: The manuscript refers to the use of Arksey and O’Malley’s scoping review framework, stating that six stages were followed. However, the original framework includes five stages. Could the authors clarify whether an additional stage was added, and if so, what it entailed? It would be helpful to explicitly state any adaptations made to the original framework to enhance methodological transparency.

**Line 73:** The authors indicate that Stage 1 of Arksey and O’Malley’s framework (identifying the research question) was followed, yet the specific research question guiding the review does not appear to be stated. For clarity and coherence, I recommend explicitly including the research question that informed the scope and direction of the review.

**Lines 83-85 :** The manuscript states that the review focused on outbreaks “potentially linked to VAW”. It is unclear how this criterion was defined or operationalized during study selection. Could the authors clarify how the link to violence against women was assessed or applied in determining which outbreaks to include or exclude? Greater transparency on this point would strengthen the rationale for the selection of cases.

**Screening**

**Lines 89-93:** The description of the screening process lacks clarity and appears inconsistent. The manuscript states that "double screening by multiple authors took place at two different stages of the review," specifically noting that 32 papers were double screened at the title and abstract stage, and 4 at full text. However, this is difficult to reconcile with the reported exclusion of 3,307 records at the title and abstract stage (as seen in PRISMA figure). Could the authors clarify exactly which stages were double screened, how these 32 and 4 papers were selected for double screening, and whether any inter-rater reliability or consistency checks were performed? How were conflicts resolved? A more detailed explanation would improve transparency and rigor.

**Data extraction**

**Lines 97-98:** The statement that "data were extracted based on the inclusion criteria to cover both supply and demand side topics using Excel" lacks sufficient detail. It is unclear what specific data were extracted from the included studies, what the extraction process entailed, how it differed for quantitative vs. qualitative studies, and how it aligned with the review objectives. Could the authors clarify what variables or information were extracted (other than identifying study information like titles, years, countries), whether a standardized extraction form was used, and how the data informed the thematic synthesis? Providing this information would enhance transparency and help readers understand the foundation of the analysis.

**Synthesis**

**Line 101:** The manuscript states that a thematic synthesis approach was used to analyze the data. However, there is no information provided on the specific steps undertaken in the analysis. As there are multiple approaches to thematic synthesis (especially in a scoping review vs. systematic reviews), it would be helpful for the authors to cite a methodological reference that guided their approach especially as the results appear to be categorical rather than thematic. As the results appear to be descriptive rather than interpretive, a descriptive approach might be more appropriate. Additionally, more detail is needed on what data were analyzed and synthesized (primary constructs? secondary constructs?), how the thematic analysis was conducted, who conducted it, which software was used, and how themes were identified and refined. Similarly, authors need to report the synthesis process (synthesizing qualitative findings with quantitative findings, primary studies’ interpretations, etc.) Also, what quality control mechanisms were used to ensure the credibility of findings? This information is essential for assessing the rigor and reproducibility of the synthesis.

**RESULTS**

**Overall**

While direct quotations from primary studies are included in some parts of the results, they are not used consistently across all themes. I encourage the authors to incorporate illustrative quotations throughout the results section to support the thematic analysis (if this was indeed the type of analysis performed, as the manuscript is missing information in the Synthesis section) and enhance the credibility and depth of the findings.

In several instances, the manuscript refers to country-level findings using broad phrases such as “South African providers reported”, “one study in Nepal”, “In Lebanon...”. However, it is unclear (from both text and Table 2) whether these statements reflect national-level experiences or the specific contexts in which the original studies were conducted (e.g., within an NGO, a government program, or a particular region). I recommend clarifying whether the various findings are drawn from individual study contexts rather than presented as representative of the entire country, to avoid overgeneralization. Instead of naming the countries in which the findings were observed, I recommend providing information about the study context, aim, participants, etc. to better contextualize the findings. I feel like the country is less relevant than the specific context in which the study was conducted (refugee camp, favelas, etc.) In other words, instead of focusing on the country, I would advise focusing on the context and reach of the study, like it was done for reference #35 (lines 197-201 “In a descriptive study […]”).

**Theme 2a Patterns:**  The theme titled “Patterns of survivor presentation for violence at health services” feels overly broad. It may be more accurate and helpful to name the specific categories within the theme itself (naming what are the patterns).

**Theme 2b Women’s experience:** As with Theme 2a (“Patterns”), the title of this theme (“Women’s experiences…”) does not clearly convey its core content. I recommend revising the theme title to more directly reflect the main finding or insight it represents, so that readers can immediately grasp its relevance and focus (e.g., **what are** the experiences of women?).

**DISCUSSION**

The stated aim of the scoping review is to identify opportunities for strengthening VAW health services during outbreaks in LMICs. However, this objective does not appear to be fully addressed in the current analysis, beyond a general call for more research. I recommend either revisiting the discussion to more explicitly identify and reflect on potential opportunities emerging from the findings, or reframing the study aim to better align with the scope and outcomes of the review.

The discussion presents findings at a general level, yet the LMIC contexts included in the review are highly diverse in terms of pandemic impact, existing health infrastructure, cultural norms, and how gender-based violence and VAW is understood and addressed. Grouping these contexts together without acknowledging this heterogeneity may oversimplify important contextual differences. I suggest addressing this point in the discussion or the limitations section, with reflection on how these variations may have influenced both the experiences reported and the generalizability of the findings.

The authors note that all included studies focused on experiences during the COVID-19 pandemic. It would be helpful to discuss why no studies were found on VAW service delivery during other outbreaks, especially given the “calls over the previous decade” (line 307) to ensure continuity of essential health services for women during such crises, and “longstanding consensus that life-saving services for survivors of violence need to be provided during outbreaks”. I am surprised to read that despite this consensus, there are no studies addressing this topic.

This might also be addressed in the synthesis section in the Methodology, but I am wondering about the inclusion of diverse study designs that capture both the perspectives of VAW service users (survivors) and service providers. While including both viewpoints is valuable, it would be important to explicitly acknowledge and discuss how these perspectives may differ or converge, even if they ultimately point to similar conclusions. Exploring these dynamics could add depth to the analysis and strengthen the interpretation of findings.

While this may fall slightly outside the scope of the study, it would be valuable to reflect on whose voices are represented in the included literature. Specifically, are the studies led by researchers based in LMICs, or primarily by teams from non-LMIC contexts? Considering this could offer important insight into how power dynamics and positionality might influence the interpretation and framing of findings.

**MINOR COMMENTS**

**OVERALL**

I recommend that authors take the time to review the manuscript, as I spotted multiple punctuation errors, and some grammar mistakes as well.

**METHODOLOGY**

Search strategy

**Line 81** “between 2014 to May 2024”: Add the month for 2014 as well.

**Lines 81-83**: Specify that this timeframe also captures the COVID-19 outbreak (in addition to Ebola 2014-2016 and Zika 2015-2016)

**Table 1 population(s) “LMICs”:** Please specify how LMICs are defined or which classification was used to defined LMICs.

**Abstract, line 112 and PRISMA figure:** The number of included articles varies (N=32 in abstract and PRISMA, N=34 in text line 112). To be standardized.

**RESULTS**

**Table 2:** Authors identified some papers with an asterisk as “key papers”, but I cannot find information about what this does imply (key for what)? Please specify.

**Table 2:** I recommend adding more details about the design of studies instead of simply naming “qualitative” “mixed” etc. Sometimes “interviews” are specified for qualitative studies, sometimes, no details are provided. Adding more details would help readers better understand the scope and reach of each study. Please also make sure to review how the data is presented (e.g., capitalized or not).

**Table 2:** I highly recommend adding the number of participants (N) for each study to help readers better grasp the scope of the studies.

**Table 2:** It would be helpful to provide more information on the context of each study (e.g., aim of study, population, etc.)

**Theme 1a. Disruptions**

**Line 28: “**were also closed in non-humanitarian during”: A word seems to be missing.

**Lines 34-36** “In Lebanon high rates […]”: This sentence is unclear (run-on) and grammatically incorrect – please address.

**Lines 36-41:** The sentences beginning with “In Kenya, Uganda […]” and “In Kenya, health workers […]” appear to convey overlapping information. I recommend revising for concision by combining or streamlining these sentences to avoid redundancy.

**Lines 57-61, l**ast paragraph of the Disruption theme: It is unclear how this paragraph fits within the theme of “disruption of services.” If the intention is to highlight services that continued or adapted successfully despite disruptions, I suggest reframing the paragraph to reflect that explicitly. It would also be helpful to briefly explain the factors that enabled these services to thrive in contrast to those that were disrupted.

**Theme 1b. Adaptation**

**Line 68:** Please define the concept of “gender digital divide”.

**Line 119:** The connection between the 2020 Beirut explosion and the actions of the GBV taskforce is unclear. Is the explosion mentioned to highlight the compounded challenges Lebanon faced, or did it directly influence the adaptations described? Clarifying this connection would help strengthen the coherence of the paragraph and its relevance to the theme of service adaptations.

**Theme 1c. Continuation**

Because paragraphs “In Brazil, primary […]” (line 147) and “Referral by health providers was […]” (line 154) both address the referral issue, I would advise combining both paragraphs into one. Another paragraph could be made for the point about the “perception that counselling was for ‘mad’ people” and for the non-existence of services regardless of COVID-19.

**Theme 2b. Women’s experiences**

**2^nd^ paragraph, line 227:** I recommend answering the first sub-objective (“describe the prevalence”) by reporting the prevalence **before** reporting the type of help sought (family, friends, neighbours, etc.)

**CONCLUSION**

Line 364: Please add “COVID-19” to specify which pandemic.
