# Supplementary material for: Health service responses and help-seeking for women experiencing violence during outbreaks in low- and middle-income settings: A scoping review
Source: PLOS Glob Public Health. 2026 Apr 30;6(4):e0004482. doi: 10.1371/journal.pgph.0004482 (PMC13132175; doi:10.1371/journal.pgph.0004482)
Supplement: Attachment — Submitted filename: instruction report.docx [file pgph.0004482.s008.docx]

Article instruction report

**Background**

Line 43: This information does not provide insight into the situation of violence against women before the pandemic. How is this proportion linked to COVID 19?

Line 44: Be specific when you talk about recent epidemics. Which epidemics are you referring to? What do you mean by recent epidemics?

Lines 47, 48, 49: You are making claims without evidence and without specifying cases, which makes your ideas debatable.

Line 59: except that the authors do not say what was done in developed countries during pandemics. Violence against women does not seem to me to be specific to low- or middle-income countries, but rather linked to gender relations and exacerbated by patriarchy. It can be seen as a determining factor in male domination. Considering this state of affairs, the context you describe seems less relevant to me.You need to problematize your subject matter more.

Line 59: *It remains to be understood...* Is this a research objective? The empirical gap that justifies the research? The scientific gap?

Lines 61, 66, 68: focus on the research objectives. I do not understand the need to formulate an objective in line 61 and others in lines 66 and 68. In principle, an article has a single objective! If you have two objectives, then you have two articles.

Line 62: This is an elements of methodology in the context

Line 66: I believe you have more than two objectives in reality:

**to examine whether (1) and to what extent health services for VAW have been delivered during recent outbreaks (2) and to identify the challenges (3) and facilitators in service provision (supply side) (4);** For this first objective, you actually have four objectives.

Lines 68,69,70: ii) **to understand how women sought help for VAW during outbreaks, (1) including the sources of support they tried to access within both the community (2) and health system (demand side) (3).** For this second objective, you have three objectives. I suggest you rephrase your objective into a single one.

**Methods**

**Search strategy and inclusion and criteria**

Line 77, It is acknowledged that the utilization of all databases is not feasible; however, this limitation constitutes a significant deficiency in this search strategy. In low-income countries, a number of studies have not been published in the selected databases.

Moreover, the rationale behind the selection of these particular database remains opaque.

Line 86, I don't think excluding populations in high-income countries is relevant. It allows you to triangulate the information and prove the specifics of low-income countries.

Lines 161, 162, I suggest that this work not be included in your work because if you include these studies in your sample, it will be biased.

**Results**

Line16, 31,32: Don’t use verbatim here. You are not using empirical data.

Line 69: Report on the author’s work, avoiding comments made by survey participants.

Line155: no specific to vaw

**General comments**

The authors discuss several pandemics, but only COVID-19 is mentioned. If there is no data on other pandemics, it would be appropriate to limit the research to COVID-19.

Furthermore, the fact that the research covers several cases limits your results. It is preferable to limit your work to a single case so that it contributes more to science. The current analysis is superficial and omits many situations. Furthermore, I believe that the situations mentioned are not specific to low- and middle-income countries. In addition, I note that you are consulting documents that are not specific to violence against women.

The research is relevant overall, but for the reasons mentioned below,

I recommend that this paper be categorised as a “Major Review”
