# Supplementary material for: Health service responses and help-seeking for women experiencing violence during outbreaks in low- and middle-income settings: A scoping review
Source: PLOS Glob Public Health. 2026 Apr 30;6(4):e0004482. doi: 10.1371/journal.pgph.0004482 (PMC13132175; doi:10.1371/journal.pgph.0004482)
Supplement: Attachment — Submitted filename: Response to reviewers_PLOS GPH_06102025.docx [file pgph.0004482.s011.docx]

# Response to reviewers

# **PLOS Global Public Health, PGPH-D-25-00671** ***Health service responses and help-seeking for women experiencing violence during outbreaks in low- and middle-income settings: a scoping review***

# Reviewer 1

**Reviewer’s feedback**

This scoping review aims to identify opportunities for strengthening violence against women (VAW) health services during outbreaks in low- and middle-income countries (LMICs), using a thematic synthesis approach. The objectives are clearly stated and the search strategy is well constructed. I appreciated the authors’ transparency regarding the limitations of both their review process and the included studies. However, I have significant concerns about key aspects of the methodology and the overall contribution of the article. In particular, the analytical and synthesis processes are not described in sufficient detail to assess the validity or credibility of the findings. Although the authors refer to a “thematic synthesis approach”, the themes presented appear more categorical in nature, raising questions about the depth of the analysis. Without clarity on how themes were developed, the results are not reproducible, and the review lacks the transparency expected in rigorous scoping reviews. Additionally, the discussion remains quite superficial and does not fully address the article’s stated aim, limiting the paper’s added value to the literature. Despite these major concerns, I have provided detailed comments, both major and minor, because I believe the manuscript has potential. With substantial revisions in three key areas (1. Clarifying the analytical and synthesis methodology, 2. Aligning the presentation of results with the stated synthesis approach, and 3. Significantly deepening the discussion in line with the paper’s stated aim), I encourage the authors to consider resubmitting a new version at a later stage.

Thank you for your thorough review and helpful feedback, we have made revisions in all three areas, and these are outlined in detail below in blue text as well as in track changes in the manuscript.

**MAJOR COMMENTS**

1. **INTRODUCTION**

While the authors define VAW and cite statistics on IPV, it remains unclear whether the review focuses solely on IPV or considers other forms of VAW (e.g., sexual violence, femicide, trafficking, early/forced marriage, etc.) Given the broad use of the term VAW, it would be helpful to clearly define what types of violence are included within the scope of this review (and later on, in the included articles) and justify this focus. This clarification would improve the conceptual clarity and ensure alignment between the stated objectives and included evidence.

This has been clarified on page 3-4. “Given that health services may treat various types of violence (e.g. usually physical, sexual and psychological) we included services for all types of VAW, including violence perpetrated by partners as well as non-partners.”

**METHODOLOGY**

2. **Study Design**

- **Line 73**: The manuscript refers to the use of Arksey and O’Malley’s scoping review framework, stating that six stages were followed. However, the original framework includes five stages. Could the authors clarify whether an additional stage was added, and if so, what it entailed? It would be helpful to explicitly state any adaptations made to the original framework to enhance methodological transparency.

Thank you for correcting this typo, 5 stages were used (as per the original framework) and this has been corrected.

- **Line 73:** The authors indicate that Stage 1 of Arksey and O’Malley’s framework (identifying the research question) was followed, yet the specific research question guiding the review does not appear to be stated. For clarity and coherence, I recommend explicitly including the research question that informed the scope and direction of the review.

Thank you for this helpful observation. We agree that the framing of the research questions required clarification. Our study has one overarching objective: to synthesize evidence on both demand and supply for health services for violence against women (VAW) during outbreaks of emerging and re-emerging pathogens over the last decade.

The text has been edited on page 4.

- **Lines 83-85 :** The manuscript states that the review focused on outbreaks “potentially linked to VAW”. It is unclear how this criterion was defined or operationalized during study selection. Could the authors clarify how the link to violence against women was assessed or applied in determining which outbreaks to include or exclude? Greater transparency on this point would strengthen the rationale for the selection of cases.

Thank you for this helpful suggestion. We agree that the original phrasing was unclear. We have now clarified in the methods section how we operationalized “potentially linked to VAW” when selecting outbreaks. The text has been edited and now reads:

“We focussed on large-scale outbreaks that disrupted the health system*, restricted access to services, and that were linked to increased risks of VAW through mobility restrictions, the impact on women’s reproductive health and sexuality, and increased economic stress within households*. *On this basis, we selected Ebola, Zika, and COVID-19, as these outbreaks meet all of these criteria and have been shown in prior research to exacerbate the risks of VAW and barriers to accessing care.”*

3. **Screening**

- **Lines 89-93:** The description of the screening process lacks clarity and appears inconsistent. The manuscript states that "double screening by multiple authors took place at two different stages of the review," specifically noting that 32 papers were double screened at the title and abstract stage, and 4 at full text. However, this is difficult to reconcile with the reported exclusion of 3,307 records at the title and abstract stage (as seen in PRISMA figure). Could the authors clarify exactly which stages were double screened, how these 32 and 4 papers were selected for double screening, and whether any inter-rater reliability or consistency checks were performed? How were conflicts resolved? A more detailed explanation would improve transparency and rigor.

This has now been edited with more detail added:

*“One author (RB) screened all abstracts and potentially eligible full text articles and extracted the data. Double screening by two co-authors (MC and JS) took place selectively at two different stages of the review, specifically for papers where there was some uncertainty over inclusion. At the title and abstract stage, two reviewers (MC and JS) double-screened 32 of papers; and 4 papers at the full text screening stage. Regular discussions between the first author and co-authors throughout the screening process ensured consistency in applying inclusion and exclusion criteria and a consensus on final decisions.”*

Inter-rater reliability checks were not performed, as this is not a standard step within the Arksey and O’Malley scoping review framework, which emphasises mapping the breadth of existing available literature rather than measuring agreement quantitatively.

4. **Data extraction**

- **Lines 97-98:** The statement that "data were extracted based on the inclusion criteria to cover both supply and demand side topics using Excel" lacks sufficient detail. It is unclear what specific data were extracted from the included studies, what the extraction process entailed, how it differed for quantitative vs. qualitative studies, and how it aligned with the review objectives. Could the authors clarify what variables or information were extracted (other than identifying study information like titles, years, countries), whether a standardized extraction form was used, and how the data informed the thematic synthesis? Providing this information would enhance transparency and help readers understand the foundation of the analysis.

This is answered below under ‘synthesis’.

5. **Synthesis**

- **Line 101:** The manuscript states that a thematic synthesis approach was used to analyze the data. However, there is no information provided on the specific steps undertaken in the analysis. As there are multiple approaches to thematic synthesis (especially in a scoping review vs. systematic reviews), it would be helpful for the authors to cite a methodological reference that guided their approach especially as the results appear to be categorical rather than thematic. As the results appear to be descriptive rather than interpretive, a descriptive approach might be more appropriate. Additionally, more detail is needed on what data were analyzed and synthesized (primary constructs? secondary constructs?), how the thematic analysis was conducted, who conducted it, which software was used, and how themes were identified and refined. Similarly, authors need to report the synthesis process (synthesizing qualitative findings with quantitative findings, primary studies’ interpretations, etc.) Also, what quality control mechanisms were used to ensure the credibility of findings? This information is essential for assessing the rigor and reproducibility of the synthesis.

Thank you for this comment. We have revised the methods section to provide more details on the data extraction and synthesis process. Specifically, the following additional description has been added:

“*Findings from the papers were extracted initially using the (deductive) themes of ‘supply’ and ‘demand’ and subsequently divided inductively into sub-themes using excel. This analysis was initially based on the themes emerging from the five papers listed as ‘key papers’ because of their primary focus on our research questions, and was then extended to the remaining papers. This process was iterative and sub-themes were refined throughout the analysis process through regular team meetings (with RB leading the analysis). This broadly followed Thomas and Harden’s [14] approach, adapted to the synthesis of secondary constructs (e.g. the authors’ interpretations of barriers/facilitators to service access) from both quantitative and qualitative data*.”

In the results, we present the findings using this thematic synthesis approach, where we present 3 emerging sub-themes for the supply side, and three sub-themes that emerged related the demand side (survivors’ help-seeking). We hope these clarifications improve transparency and make the synthesis process clearer for readers.

As mentioned on page 9:

“We did not conduct a quality assessment of the included studies, in line with scoping review methodology, which focuses on mapping the breadth of the literature rather than assessing the quality of individual study findings.”

6. **RESULTS**

- **Overall**
- While direct quotations from primary studies are included in some parts of the results, they are not used consistently across all themes. I encourage the authors to incorporate illustrative quotations throughout the results section to support the thematic analysis (if this was indeed the type of analysis performed, as the manuscript is missing information in the Synthesis section) and enhance the credibility and depth of the findings.

Thank you for the comment. Where quotations were provided in the included studies, we have used them selectively to illustrate particular points, but they were not available across all themes. We have clarified this in the methods (page 7):

“Since this was a thematic synthesis of secondary constructs, participant quotes are only included to illustrate authors findings where these were presented in the original studies, and as such they are not included for all themes.”

- In several instances, the manuscript refers to country-level findings using broad phrases such as “South African providers reported”, “one study in Nepal”, “In Lebanon...”. However, it is unclear (from both text and Table 2) whether these statements reflect national-level experiences or the specific contexts in which the original studies were conducted (e.g., within an NGO, a government program, or a particular region). I recommend clarifying whether the various findings are drawn from individual study contexts rather than presented as representative of the entire country, to avoid overgeneralization. Instead of naming the countries in which the findings were observed, I recommend providing information about the study context, aim, participants, etc. to better contextualize the findings. I feel like the country is less relevant than the specific context in which the study was conducted (refugee camp, favelas, etc.) In other words, instead of focusing on the country, I would advise focusing on the context and reach of the study, like it was done for reference #35 (lines 197-201 “In a descriptive study […]”).

Thank you for this suggestion, this has been edited throughout where relevant. Including on page 17:

“In Lebanon a qualitative study with GBV and humanitarian key informants reported high rates of staff burnout, making it challenging to sustain GBV service provision, and staff safety concerns were also high with threats and theft reported by most service providers [26].”

Some studies included national level data collection and this is now mentioned in the summary table (Table 2) including the target population.

- **Theme 2a Patterns:**  The theme titled “Patterns of survivor presentation for violence at health services” feels overly broad. It may be more accurate and helpful to name the specific categories within the theme itself (naming what are the patterns).

We have edited this to:*“Increases and decreases* in survivor*s* presenting at health services”

- **Theme 2b Women’s experience:** As with Theme 2a (“Patterns”), the title of this theme (“Women’s experiences…”) does not clearly convey its core content. I recommend revising the theme title to more directly reflect the main finding or insight it represents, so that readers can immediately grasp its relevance and focus (e.g., **what are** the experiences of women?).

Thank you for the comment. The title of 2.b. is “Women’s experiences seeking help with informal sources such as community members and family”.

We feel that this is already quite explicit as to the content covered under this heading.

7. **DISCUSSION**

- The stated aim of the scoping review is to identify opportunities for strengthening VAW health services during outbreaks in LMICs. However, this objective does not appear to be fully addressed in the current analysis, beyond a general call for more research. **I recommend either revisiting the discussion to more explicitly identify and reflect on potential opportunities emerging from the findings**, or reframing the study aim to better align with the scope and outcomes of the review.

Thank you for this comment. We would like to clarify that the purpose of the review was indeed to identify opportunities for strengthening VAW health services during outbreaks in LMICs, and the entire analysis is structured around identifying both gaps and opportunities in the existing evidence base. This focus is reflected throughout the discussion, where we highlight challenges continuing care, and the opportunities but also shortcomings of adaptions made by service providers. We have therefore not edited the discussion beyond the edits outlined under subsequent comments.

- The discussion presents findings at a general level, yet the LMIC contexts included in the review are highly diverse in terms of pandemic impact, existing health infrastructure, cultural norms, and how gender-based violence and VAW is understood and addressed. Grouping these contexts together without acknowledging this heterogeneity may oversimplify important contextual differences. I suggest addressing this point in the discussion or the limitations section, with reflection on how these variations may have influenced both the experiences reported and the generalizability of the findings.

We agree, and as with many scoping reviews the large number of papers we identified covered many different types of settings and captured significant heterogeneity in experiences during COVID-19, health systems and norms around VAW. We have added the follow text to the discussion:

“Included papers were diverse in terms of study design, regions where research was conducted, experiences of lockdowns, and existing health infrastructure. Paper also differed in the inclusion of both perspectives on health service provision (these papers often drew on data collected from providers or from facilities) and access to health services (these papers often drew on data collected from service users). However a consistent picture emerged showing there were significant challenges for women to access health care after facing violence.”

- The authors note that all included studies focused on experiences during the COVID-19 pandemic. It would be helpful to discuss why no studies were found on VAW service delivery during other outbreaks, especially given the “calls over the previous decade” (line 307) to ensure continuity of essential health services for women during such crises, and “longstanding consensus that life-saving services for survivors of violence need to be provided during outbreaks”. I am surprised to read that despite this consensus, there are no studies addressing this topic.

We were also surprised, however the fact that we did not find papers from other outbreaks demonstrates that this remains a neglected topic for primary research outside of the COVID-19 pandemic.

We have added some text to the discussion reflecting on why this might be (page 27)

*This increase in research interest may have partly been triggered by the widespread public attention to intimate partner violence (initially in high income settings) during the COVID-19 lockdowns, when women were forced to stay at home with violent partners.*

And we have already mentioned the research gap on other (non-COVID-19) outbreaks on page 27:

*“There is a significant research gap in experiences from the range of other (non-COVID-19) outbreaks that have also shattered heath systems and exacerbated violence. This gap is striking considering that outbreak responses to Ebola (in West Africa and Democratic Republic of the Congo) and Zika (in the Americas) have also been critiqued as gender blind and as having neglected other essential services for women [51–54].”*

- This might also be addressed in the synthesis section in the Methodology, but I am wondering about **the inclusion of diverse study designs that capture both the perspectives of VAW service users (survivors) and service providers**. While including both viewpoints is valuable, it would be important to explicitly acknowledge and discuss how these perspectives may differ or converge, even if they ultimately point to similar conclusions. Exploring these dynamics could add depth to the analysis and strengthen the interpretation of findings.

Thank you for this comment, as with many scoping reviews, we did find papers with a range of study designs and with a range of different types of participants.

In the discussion (page 28) we have added the following text:

“Included papers were diverse in terms of study design, regions where research was conducted, experiences of lockdowns, and existing health infrastructure. Papers also differed in the inclusion of both perspectives on health service provision (these papers often drew on data collected from providers or from facilities) and access to health services (these papers often drew on data collected from service users). However a consistent picture emerged showing there were significant challenges for women to access health care after facing violence.”

- While this may fall slightly outside the scope of the study, it would be valuable to reflect on whose voices are represented in the included literature. Specifically, are the studies led by researchers based in LMICs, or primarily by teams from non-LMIC contexts? Considering this could offer important insight into how power dynamics and positionality might influence the interpretation and framing of findings.

Thank you for your valuable suggestion. LMIC researchers appear to be well represented in the literature with many of the papers first authored by researchers whose institution indicates they are likely to be based in the setting where the study was conducted (although this is an assumption). We have now mentioned this on page 11:

“First authors from LMIC institutions where the research was conducted appear to be well represented.”

**MINOR COMMENTS**

8. **OVERALL**

- I recommend that authors take the time to review the manuscript, as I spotted multiple punctuation errors, and some grammar mistakes as well.

This has now been reviewed.

9. **METHODOLOGY**

**Search strategy**

- **Line 81** “between 2014 to May 2024”: Add the month for 2014 as well.

This has been corrected.

- **Lines 81-83**: Specify that this timeframe also captures the COVID-19 outbreak (in addition to Ebola 2014-2016 and Zika 2015-2016)

This has been corrected.

- **Table 1 population(s) “LMICs”:** Please specify how LMICs are defined or which classification was used to defined LMICs.

We relied on the lists of LMIC countries provided by the databases at the time of our search (e.g. the expert searches on Medline and Embase).

We have added the following:

“The search was limited to studies conducted in LMICs *(as defined by each of the databases at the time of our search)* between January 2014 to May 2024*.”*

- **Abstract, line 112 and PRISMA figure:** The number of included articles varies (N=32 in abstract and PRISMA, N=34 in text line 112). To be standardized.

This typo has been corrected (page 6).

10. **RESULTS**

- **Table 2:** Authors identified some papers with an asterisk as “key papers”, but I cannot find information about what this does imply (key for what)? Please specify.

An explanation is now provided:

“Most studies provided only minimal findings directly relevant to on our research topic….While these studies included some incidental data on VAW health service access, only five articles had this as their primary focus (*these are marked as ‘key papers’ in Table 2*) [23,27,28,31,41].”

- **Table 2:** I recommend adding more details about the design of studies instead of simply naming “qualitative” “mixed” etc. Adding more details would help readers better understand the scope and reach of each study.

We have now added a column to Table 2 (summary table) labelled ‘methods’ that includes a summary of the methods.

- Sometimes “interviews” are specified for qualitative studies, sometimes, no details are provided. Please also make sure to review how the data is presented (e.g., capitalized or not).

This has been corrected.

- **Table 2:** I highly recommend adding the number of participants (N) for each study to help readers better grasp the scope of the studies.

We have now added a column to Table 2 (summary table) labelled ‘methods’.

- **Table 2:** It would be helpful to provide more information on the context of each study (e.g., aim of study, population, etc.)

We have now added additional columns to Table 2 (summary table) labelled ‘study aim/objectives’ and ‘target population’.

**Theme 1a. Disruptions**

- **Line 28: “**were also closed in non-humanitarian during”: A word seems to be missing.

This has been corrected to ‘non-humanitarian settings’.

- **Lines 34-36** “In Lebanon high rates […]”: This sentence is unclear (run-on) and grammatically incorrect – please address.

This has been corrected:

In Lebanon a qualitative study with GBV and humanitarian key informants reported high rates of staff burnout, making it challenging to sustain GBV service provision, and staff safety concerns were also high with threats and theft reported by most service providers [26].

- **Lines 36-41:** The sentences beginning with “In Kenya, Uganda […]” and “In Kenya, health workers […]” appear to convey overlapping information. I recommend revising for concision by combining or streamlining these sentences to avoid redundancy.

The duplicated text has been removed.

- **Lines 57-61, l**ast paragraph of the Disruption theme: It is unclear how this paragraph fits within the theme of “disruption of services.” If the intention is to highlight services that continued or adapted successfully despite disruptions, I suggest reframing the paragraph to reflect that explicitly. It would also be helpful to briefly explain the factors that enabled these services to thrive in contrast to those that were disrupted.

This paper provides a contradictory example, however as it is only one example we have decided to keep the thematic heading focussed on ‘disruptions’.

**Theme 1b. Adaptation**

- **Line 68:** Please define the concept of “gender digital divide”.

The following has been added:

“Safety concerns were the most pressing ethical consequence, and the gender digital divide, with significant disparities in digital access and benefits between women and men. “

- **Line 119:** The connection between the 2020 Beirut explosion and the actions of the GBV taskforce is unclear. Is the explosion mentioned to highlight the compounded challenges Lebanon faced, or did it directly influence the adaptations described? Clarifying this connection would help strengthen the coherence of the paragraph and its relevance to the theme of service adaptations.

Some edits have been made:

“In Lebanon, the pandemic overlapped with multiple crises including the 2020 Beirut explosion compounding challenges for providers. In terms of adaptations, the (national) GBV taskforce implemented harmonised guidance and tools for actors at field level to adapt to remote modalities for GBV services (e.g. training on remote case management)[28].

**Theme 1c. Continuation**

- Because paragraphs “In Brazil, primary […]” (line 147) and “Referral by health providers was […]” (line 154) both address the referral issue, I would advise combining both paragraphs into one. Another paragraph could be made for the point about the “perception that counselling was for ‘mad’ people” and for the non-existence of services regardless of COVID-19.

These paragraphs are now grouped together.

**Theme 2b. Women’s experiences**

- **2^nd^ paragraph, line 227:** I recommend answering the first sub-objective (“describe the prevalence”) by reporting the prevalence **before** reporting the type of help sought (family, friends, neighbours, etc.)

For simplicity this has not been edited.

11. **CONCLUSION**

- Line 364: Please add “COVID-19” to specify which pandemic.

This has been corrected.

Reviewer 2

Article instruction report

1. **Background**

- Line 43: This information does not provide insight into the situation of violence against women before the pandemic. How is this proportion linked to COVID 19?

This reference (Kifle et al.) is simply cited to show that VAW was highly prevalent during COVID-19, in the following paragraphs we outline the need for specific health system responses during outbreaks given there are unique challenges during these periods.

- Line 44: Be specific when you talk about recent epidemics. Which epidemics are you referring to? What do you mean by recent epidemics?

Based on the publications referenced in this sentence we have now mentioned Ebola and COVID-19.

- Lines 47, 48, 49: You are making claims without evidence and without specifying cases, which makes your ideas debatable.

References have been added to this sentence: “During outbreaks, essential health services for women, including those for maternal, and SRH, are often deprioritised or disrupted [7,8].”

- Line 59: except that the authors do not say what was done in developed countries during pandemics. Violence against women does not seem to me to be specific to low- or middle-income countries, but rather linked to gender relations and exacerbated by patriarchy. It can be seen as a determining factor in male domination. Considering this state of affairs, the context you describe seems less relevant to me. You need to problematize your subject matter more.

We fully agree that violence against women is a global problem and not confined to LMIC settings. Global VAW estimates do show that higher prevalence rates in LMIC countries, but, as you note, the root causes of VAW (e.g. patriarchal gender relations) are universal. We would like to clarify that our focus is not on the drivers of VAW, but instead on how health system and service responded to VAW during outbreaks. We have revised the text on the study objectives to make this distinction clearer.

In addition, we have expanded our discussion (page 27) to mention the increased attention (initially) in high income settings to the topic of IPV during the COVID-19 lockdowns:

“Although our search focussed on multiple recent outbreaks (Ebola, Zika, COVID-19), all 32 papers included only examined experiences during COVID-19. This increase in research interest may have partly been triggered by the widespread public attention to intimate partner violence (initially in high income settings) during the COVID-19 lockdowns, when women were forced to stay at home with violent partners.”

- Line 59: *It remains to be understood...* Is this a research objective? The empirical gap that justifies the research? The scientific gap?

This is an evidence gap. The sentence has now been edited for clarity:

‘Whilst normative efforts (e.g. policy briefs) on how to address VAW have accelerated since the COVID-19 pandemic [8,9], there remains an evidence gap from low-and-middle-income settings on what health service responses have been implemented and women’s help-seeking in the context of outbreaks [10]’.

- Lines 61, 66, 68: focus on the research objectives. I do not understand the need to formulate an objective in line 61 and others in lines 66 and 68. In principle, an article has a single objective! If you have two objectives, then you have two articles.

Thank you for this helpful observation. We agree that the framing of the objectives required clarification. Our study has one overarching objective: to synthesize evidence on both demand and supply for health services for violence against women (VAW) during outbreaks of emerging and re-emerging pathogens over the last decade.

In the draft you have reviewed, we described this overarching objective in two parts (supply and demand), which may have given the impression of multiple objectives. To avoid confusion, we have now revised the text to present a single overarching objective, accompanied by two sub-questions: (i) health service delivery (supply side), and (ii) women’s help-seeking (demand side). This revision clarifies that the article is unified under one objective, while still allowing us to examine these two interrelated dimensions.

- Line 62: This is an elements of methodology in the context

The sentence you refer to has been moved from context to the methods section (page 5):

*“*We included articles on healthcare, health policy and community health responses, as well as survivors’ help-seeking behaviours.”

- Line 66: I believe you have more than two objectives in reality: to examine whether (1) and to what extent health services for VAW have been delivered during recent outbreaks (2) and to identify the challenges (3) and facilitators in service provision (supply side) (4); For this first objective, you actually have four objectives. Lines 68,69,70: ii) to understand how women sought help for VAW during outbreaks, (1) including the sources of support they tried to access within both the community (2) and health system (demand side) (3). For this second objective, you have three objectives. I **suggest you rephrase your objective into a single one.**

Please see our reply above, and the edits made under Lines 61,66 and 68 to rephrase the objectives.

2. **Methods**

**Search strategy and inclusion and criteria**

- Line 77, It is acknowledged that the utilization of all databases is not feasible; however, this limitation constitutes a significant deficiency in this search strategy. In low-income countries, a number of studies have not been published in the selected databases. Moreover, the rationale behind the selection of these particular database remains opaque.

Thank you for the comment. We selected Medline, Embase, Global Health, and Global Index Medicus to capture a breadth of literature across biomedical, public health, and global health domains, with a particular focus on low- and middle-income settings.

- **Medline** was included as a core biomedical database with strong coverage of peer-reviewed literature on health services and public health.
- **Embase** complements Medline by providing broader international coverage, especially in public health, health systems, and health service delivery, and by indexing more journals from outside North America.
- **Global Health** was chosen because of its specialist focus on international and public health, including social, policy, and health systems research that is often underrepresented in other databases.
- **Global Index Medicus (GIM)** was included to ensure representation of literature from low- and middle-income countries, as it collates regional databases maintained by the WHO thereby capturing research outputs that may not be indexed in traditional biomedical databases.

The following text has been added to page 4:

*“These databases were selected to capture a breadth of literature across public health and global health domains with a particular focus on LMIC literature.”*

- Line 86, I don't think excluding populations in high-income countries is relevant. It allows you to triangulate the information and prove the specifics of low-income countries.

One of our exclusion criteria was populations in high income countries. Our review explicitly focussed on LMIC settings because outbreaks in these contexts often present unique health system challenges, including constrained resources, fragile infrastructures, and limited access to essential services. These conditions differ significantly from those in high-income countries. We thought that including evidence from high-income settings, where health systems are generally more resilient, could obscure the context-specific barriers and responses that are most relevant to LMICs.

While we agree that triangulation across contexts can indeed offer valuable insights, our objective was to map the literature specific to LMICs, where the need for targeted guidance is greatest and where existing literature is comparatively limited. We have clarified this rationale in the manuscript to make explicit why LMICs were prioritised as the focus of this review.

- Lines 161, 162, I suggest that this work not be included in your work because if you include these studies in your sample, it will be biased.

We have retained these studies because they included data collected during the pandemic and provide important insights into women’s use of informal support services. In the results section 2.b., we clarify which patterns reflect pre-pandemic trends and which describe pandemic-specific disruptions, to reduce the risk of bias while reflecting the breadth of available evidence.

**Results**

- Line16, 31,32: Don’t use verbatim here. You are not using empirical data.

Thank you for the comment. We agree that our review is based on secondary constructs and not primary data. In order to clarify this, we have revised the methods (page 7) to clarify that “*we extracted and synthesized secondary constructs (e.g. the authors’ interpretations of barriers/facilitators to service access) rather than any primary quantitative or qualitative data.”*

The participant quotes in our results are those that are reported in the original studies and are used selectively in our results section to illustrate authors’ findings.

- Line 69: Report on the author’s work, avoiding comments made by survey participants.

This has now been edited to:

“In Kenyan and South African study sites, authors reported that counselling services were provided remotely to survivors during the lockdowns [31].”

- Line155: no specific to vaw

We agree that various health services are limited or non-existent in some LMIC settings however we have focussed here on what papers reported on VAW services, rather than a broader range of services.

3. **General comments**

- The authors discuss several pandemics, but only COVID-19 is mentioned. If there is no data on other pandemics, it would be appropriate to limit the research to COVID-19.

Thank you for your comments, indeed we did search for papers from Zika, Ebola and COVID-19. It is important to highlight that *no papers* were found (meeting our inclusion criteria) from the other outbreaks.

- Furthermore, the fact that the research covers several cases limits your results. It is preferable to limit your work to a single case so that it contributes more to science. The current analysis is superficial and omits many situations. Furthermore, I believe that the situations mentioned are not specific to low- and middle-income countries. In addition, I note that you are consulting documents that are not specific to violence against women. The research is relevant overall, but for the reasons mentioned below, I recommend that this paper be categorised as a “Major Review”

Thank you for the comment. All papers included in the review reported on VAW health service provision or use during outbreaks as per our inclusion and exclusion criteria, as we noted under ‘characterisation of studies’:

*“Most studies provided only minimal findings directly relevant to our research topic. Instead, they primarily focussed on issues such as increased rates of VAW during the pandemic or the impact of the pandemic on mental health or/and SRH services.* *While these studies included some incidental data on VAW health service access, only five articles had this as their primary focus (these are marked as ‘key papers’ in Table 2) [23,27,28,31,41].”*

As we have mentioned in the discussion and findings, although our search included other outbreaks (Ebola and Zika) we only found papers that focussed on COVID-19. We agree that VAW during outbreaks is not unique to LMIC settings, however we limited our search to LMIC settings because the health system constraints in these settings are significantly different to those in HICs and our aim was to generate evidence that is most relevant to LMICs health responses.

# Reviewer 3

**Overall assessment:**

This study shines a light on a reportedly underexplored aspect of the literature the impacts

of disease outbreaks on the impact the provision of and access to health services for

violence against women. The authors provide a much-needed description on the published

primary studies addressing this topic in the English language, succinctly highlighting the

major takeaways from the studies included. The paper includes a very helpful breakdown

of the key themes in the findings reported by authors in a number of settings in LMICs and

serves to highlight the considerations for future research, interventions, and advocacy.

Some minor changes are needed to improve the manuscript and prevent any

misinterpretations of the findings reported.

- Harmonization of the language surrounding the forms of sexual violence is warranted. One suggestion would be to define all the acronyms describing the various forms of violence (GBV, VAW, SGBV) in one spot – briefly discussing the ambiguity and overlap between those concepts.

Within the findings we have kept the language as per the original papers included. However, under search strategy and inclusion criteria (page 5) we have added the following clarification on terms:

“*There are several terms that are often used interchangeably (and sometimes ambiguously) in the literature to describe violence experienced by women and other populations. ‘Gender-based violence’ (GBV) or ‘sexual and gender-based violence’ describe any harmful act directed at a person because of their (socially prescribed) gender, GBV is often (but not exclusively) perpetrated against women. As part of examining violence against women we also included intimate partner violence, which is one of the most prevalent forms of VAW.”*

*The inclusion criteria (Table 1) has also been edited and now reads:*

*“women survivors of VAW, gender-based violence (GBV) or sexual and gender-based violence (SGBV).”*

1. **General comments**

- I would caution the authors against slipping from language reporting the findings of the

scoping review to statements reporting on the success of certain interventions. Given that no assessment of the quality of the studies was conducted, authors should beware of attributing success to the interventions described. In the results section, the authors write on line 127-128 “the approach was successful in serving some harder to reach women”. Such statements should be reported carefully. For example, In the results section, lines 5-6: this scoping review isn’t so much reporting women’s help-seeking behavior during the pandemic, as it is highlighting **what the literature on VAW reports** when it comes to women’s help-seeking behavior. This is an important distinction that should be made clear throughout the text. Another example is clear on line 223: “We will first describe the prevalence of help-seeking from community members…”. This sentence reads as if the authors take it for granted that the studies cited accurately reflect that prevalence of help-seeking behavior, even though no assessment of the quality of the study and the validity of their conclusions can be made. Statements like “The authors of X study reported that…” can reduce that ambiguity.

Thank you for this suggestion, this has been edited throughout, and we have now nuanced the text to clarify that findings are reported by authors in the papers we included. This included in the sections mentioned: lines 127-128 and lines 5-6, and line 223 (which have been pasted below).

*“The approach was reported by authors as successful in serving some harder to reach women”*

“*First, we present the key findings in the literature related to supply themes, focusing on health service delivery for VAW during COVID-19 (with no papers found that focussed on other outbreaks). Then, we present the key findings in the literature related to demand themes, highlighting women’s help-seeking during the pandemic.”*

“*We will first describe the prevalence of help-seeking from community members that was reported by authors (which may not be specific to the pandemic period) and then we will explore pandemic-specific disruptions to help-seeking reported from these community supports as well as survivors’ coping mechanisms*.”

Specific comments:

2. **Abstract:**

- The authors jump directly into the description of the scoping review objectives (line 15) without clearly stating how VAW are linked to disease outbreaks. One sentence after “Services may be disrupted or deprioritized, or woman may avoid clinical settings” is needed to lead into the scoping review objectives.

As our scoping review does not focus on the connection between VAW and outbreaks we have decided not to highlight this in the first sentences of the abstract considering word restrictions.

3. **Introduction**

- Line 112: The authors mention that 34 articles met the inclusion criteria and were included in the final synthesis. Yet subsequent text and figures only mention 32 articles.

This has been corrected, the number is 32.

4. **Results:**

- Line 27: Consider rephrasing “’Safe spaces’ were also closed in non-humanitarian

[*settings?*] during the pandemic…”

This has been corrected.

- Line 57: Suggested change ‘There was also **one** account of VAW interventions delivered successfully despite the pandemic….”

The original phrasing has been kept as it is more accurate, there are some success stories reported elsewhere in the papers.

- Line 59: Change to “locally-**led** solutions”

This is now corrected.

- Lines 59-60: Clearly specify that the 642% monthly increase is *reported* by the authors of the Kenya study. As no quality assessment was performed on the studies, one cannot take the reported improvements at face value with no discussion of the quality of these findings.

This has been edited as suggested.

- Line 164-165: Consider clarifying “In Ethiopia, services, for example from women’s aiairs and health facilities, were particularly limited at the local administrative (Kebel) level.”

No further information is provided from the authors of this paper.

- Line 202: Clarify the statement: “In some cases, such as cross-sectional studies, it was unclear whether the pandemic saw changes in help-seeking given that in many setting survivors rarely seek help from formal services.”

This has been edited as follows:

*“In some cases, such as a cross sectional studies, it was unclear whether the pandemic saw changes in help-seeking considering studies did not compare to service use pre-pandemic and that in many settings survivors rarely seek help from formal services regardless of outbreaks.”*
