# Supplementary material for: Health service responses and help-seeking for women experiencing violence during outbreaks in low- and middle-income settings: A scoping review
Source: PLOS Glob Public Health. 2026 Apr 30;6(4):e0004482. doi: 10.1371/journal.pgph.0004482 (PMC13132175; doi:10.1371/journal.pgph.0004482)
Supplement: Attachment — Submitted filename: Article report 2025.docx [file pgph.0004482.s010.docx]

**Article report**

**Background**

Lines: 62, 63, 64

For me, the scientific contribution of this study would have been a comparative analysis of the three types of environment. In my opinion, limiting the study to low and middle incomes does not seem justified, particularly given that the crisis was global. However, this could be a focus for your future research.

Lines 62 to 64.The authors still need to make an effort to clearly convey the objective of the research. With reference to what is proposed, I see two main objectives linked by 'and to'.

Lines 70 to 74: I think there are more than two specific questions. Check the wording.

**Search results**

**Characteristics of studies**

In the first version of the paper, I recommended focusing exclusively on COVID-19. The authors have provided explanations, but I think you need to reconsider lines 174-175. If your study addresses a gap, whether scientific or empirical, this gap cannot be relevant if you are dealing with Ebola or Zika, as there is no data available. For a study covering three epidemics, there is only data related to one epidemic: COVID-19. If you cannot remove the other two cases (Ebola and Zika) from your work, please make an effort to show the methodological implications that this entails. What methodological lesson should be learned from this? In science, I think it is much more important to understand the reasons behind the choices made.

**Results**

***1. Health service delivery for VAW during COVID-19 (supply themes)***

**1.a. Disruptions to VAW health service delivery during COVID-19**

Line 18- Despite the authors' explanations regarding the use of verbatim quotes, I believe that this method is inappropriate. I therefore reiterate my observation that the authors should remove the verbatim quotes. I reserve judgment when you include verbatim quotes in your work, which is exclusively a review of the literature. I believe that it is the results that are of interest. You can therefore do without verbatim quotes, which are not a result in themselves.

Line 27- The concept of social distancing needs to be deconstructed. It is true that it has been used in public health to determine health measures, but opt for the concept of physical distancing for greater precision and empathy.

**Discussion**

Line 293, I think it would be appropriate to use the names of the epidemics: Ebola, Zika, COVID-19, instead of the word “recent epidemics.” It is impossible to systematically date the word “recent.” Your work must stand the test of time.

**General comments**

This second version of the article seems to me to be an improvement. We felt that the choice of low- and middle-income sites was not justified and constituted a bias in the study. Improvements have been made in this regard. I note an effort to improve the work. However, I remain convinced that the scientific contribution of this work would have been significant if the authors had opted for a comparative study between low-, medium-, and high-income situations. I believe that highlighting the different specificities of each country would have been of definite scientific interest and would be of paramount importance in terms of programming. In view of the efforts made, I think this article should be accepted with minor revisions.
