# Supplementary material for: Health service responses and help-seeking for women experiencing violence during outbreaks in low- and middle-income settings: A scoping review
Source: PLOS Glob Public Health. 2026 Apr 30;6(4):e0004482. doi: 10.1371/journal.pgph.0004482 (PMC13132175; doi:10.1371/journal.pgph.0004482)
Supplement: Attachment — Submitted filename: PLOS GPH response to reviewers_18032026.docx [file pgph.0004482.s012.docx]

*PLOS GPH, second revision, response to reviewers*

*Reviewer 2*

**Reviewer #2: I would like to thank the authors for responding to my previous comments and for providing clear and thoughtful answers. Below, I offer additional remarks that I believe could further strengthen the manuscript. Most of these are minor comments and suggestions. The major issues concern the alignment between the methodology described for data analysis and the formulation of the themes and resulting findings. At present, the methodology does not fully correspond to the results, which constitutes a relatively significant methodological issue. I strongly encourage the authors to address the major recommendations provided.**

Thank you for your additional comments. Detailed replies have been provided below and can be found in track changes in the manuscript.

1. **Minor - Various locations - “women’s help-seeking for violence” seems a bit clumsy as it can sound like women are seeking help to commit violence rather than seeking help because they are experiencing violence. We recommend rephrasing (e.g., help-seeking related to violence, help-seeking behaviors in response to violence, help-seeking among women experiencing violence, etc.)**

This phrasing has been changed throughout.

2. **Minor - Various locations - Please proofread this newer version of the manuscript, there are many punctuation mistakes, for example**

- **A space is missing in “weredeprioritised” (line 48)**
- **commas are missing after “e.g.” (e.g., line 65, Table 1)**
- **There often appear to be two spaces in a row. Please correct this (e.g., line 68 and line 71 after ‘VAW’)**
- **Make sure you use em dash rather than hyphen for parenthetical thoughts (e.g., line 69 “such as Ebola […]”)**
- **Avoid using two punctuation marks following one another (e.g., line 74 ?.)**
- **I think the capitals are unnecessary in “Eighteen interviews” and “Epidemiological Surveillance” (Table 2)**
- **Most objective/aim in Table 2 ends with a period, except Decjer et al., 2022. Please standardize.**
- **Some Study periods end with a period (e.g., Vahedi et al., 2023; Yirgu et al., 2023), others don’t. Please standardize**

The above changes have been made throughout. Very minor edits to improve the written expression have been made throughout the manuscript.

**- Line 89 in the results: remove parentheses at the end of the sentence**

We feel the sentence should remain as it:

“There were greater challenges in humanitarian settings or for marginalised populations.”.

**-Commas are missing, for example in the “Similarly in Bangladesh the United […]” sentence (Results line 144) or “In another study in Kenya data was […]” sentence (Results line 178)
-“s” is missing from “health workerS strike” (line 180 Results)**
**- comma missing after “Uganda” (line 281)**

**-commas missing in sentence on lines 368-371 (Mpox limitation) and it runs two independent clauses together without proper separation. I recommend diving the sentence in two.
Other mistakes were also spotted so a thorough read would help correct them.**

These have been corrected throughout.

**3. Minor - Abstract - This sentence is confusing to read “Understand how health services for violence against women (VAW) were affected in low- and middle-income (LMIC) settings during recent outbreaks, and women’s help-seeking for violence.” Please consider rewriting it (e.g., use the phrasing used in the background section linee 62-64)**

This has been edited to:

*“We conducted a scoping review* *to understand how health services for violence against women (VAW) were affected in low- and middle-income (LMIC) settings during recent outbreaks, and to understand women’s help-seeking for violence during these periods.”*

**4. Minor - Background line 38 - Please define VAW, ideally in relation to IPV and GBV (see comment #8). It will help understand its connection with IPV, GBV and SGBV.**

We have defined the terms used (page 4):

*“Violence against women can be described as a range of acts of gender-based violence that results in, or is likely to result in, physical, sexual or mental hard or suffering (UN, 1993). In the literature there are several terms that are often used interchangeably to describe violence experienced by women, gender based violence (GBV) is an umbrella term referring to violence that can affect men, women, or LGBTI+ communities. As part of examining violence against women we also included intimate partner violence, which is one of the most prevalent forms of VAW.”*

**5. Minor - Background line 54 - Please specify if “80% of countries” concerns all countries or only LMIC**

This is global, this has been clarified.

**6. Minor- Background line 58 - I am not sure “policy briefs” (and advocacy document) can be described as “normative efforts”, which would be norms, guidelines. Please refine the wording.**

‘Policy brief’ has been removed.

**7. Minor - Methods lines 92-94 - Please add references to support this statement “[…], as these outbreaks met these criteria and have been shown in prior research to exacerbate the risks of VAW and barriers to accessing care.”**

References have been added.

**8. Minor - Methods lines 98-100 - GBV is defined, but it’s not specified whether it’s included or not in VAW. Please specify. At this point, it’s unclear to me how the concepts of GBV, VAW and IPV are related especially in this study (e.g., are VAW and IPV subtypes of GBV? Then, wouldn’t the inclusion of GBV already include VAW? But if this scoping review is focused on VAW, then it would exclude other types of GBV?)**

These terms are often used interchangeably in the literature included in this review, therefore we included papers that used the terms VAW. We have now added some definitions of different terms used in the literature on page 4:

*“Violence against women can be described as a range of acts of gender-based violence that results in, or is likely to result in, physical, sexual or mental hard or suffering [13]. In the literature there are several terms that are often used interchangeably to describe violence experienced by women, gender based violence (GBV) is an umbrella term referring to violence that can affect men, women, or LGBTI+ communities. As part of examining violence against women we also included intimate partner violence, which is one of the most prevalent forms of VAW.”*

**9. Minor - Methods Table 1 - In the population(s) row, are both cis and trans women included (would you have excluded trans women to focus on cis women)? Please specify.**

Our focus was women more broadly and all populations defined as women by authors, this appeared to be generally cis women although it was usually not specified within individual papers.

**10. Minor Methods Table 1 Please specify what “members of affected communities” mean. Affected by VAW? GBV? SGBV? Affected by the pandemic?**

This has been edited to ‘members of communities affected by outbreaks’.

**11. Minor - Methods Table 1 (Limitation section, line 365) -Focusing on concept 1 (health service search terms) may have limited the findings about women’s help-seeking patterns and behaviors (2nd objective and research question). Ideally, a second specific search strategy would have been used for the second objective and research question (excluding the 1st concept). Specifically, in the “intervention(s)” section of Table 1, only studies looking at access to, delivery of health services interventions, health outreach/awareness programs are included and only studies describing health services (“outcome(s)” row) are included. Women may have sought help outside of these formal programs/interventions, so limiting the search to this concept is a limitation to the understanding of help-seeking patterns and behaviors. Please address it in the limitation section.**

Indeed women may have sought help from various sources. However concept 1 search terms were chosen given that our focus for this specific review was on help-seeking within the health system, and we excluded help-seeking for justice, livelihood and other types of interventions.

This is covered in our objectives on page 4:

*“We conducted a scoping review of the literature to synthesise evidence on both the supply and demand for health services for VAW during outbreaks of emerging/re-emerging pathogens−­­­ such as Ebola, Zika and COVID-19­−in order to identify disruptions, adaptations, and gaps in service delivery and help-seeking.”*

**12. Minor - Methods Table 1 - Would reviews reporting primary results be included? If not, please add “reviews” in the “exclusion” column.**

Reviews reporting primary results were not excluded.

**13. Minor - Figure 1 (PRISMA) - PRISMA figure incomplete. Many numbers are missing (blank) from the PRISMA figure, including the number of articles excluded based on titles and abstracts screening, the articles sought for retrieval, article not retrieved, article assessed in full text for eligibility. Please complete the PRISMA figure**

This has now been fixed.

**14. Minor - Synthesis line 121 - “supply” and “demand” are not themes, but rather categories. Please address the vocabulary used.**

While this review follows the scoping review framework proposed by Arksey and O’Malley, the analytical approach used to synthesis findings was informed by the thematic synthesis approach by Thams and Harden (2008). On page 7 we have now detailed why we have termed these ‘themes’:

*“This thematic synthesis broadly followed Thomas and Harden’s [17] approach, adapted to the synthesis of secondary constructs (e.g. the authors’ interpretations of barriers/facilitators to service access) from both quantitative and qualitative data. Thomas and Harden’s approach includes inductive coding of findings from papers in order to develop ‘descriptive’ themes, and following this the development of ‘analytical’ themes where the reviewers go beyond the individual studies to develop new constructs or explanations.”*

**15. Minor - Synthesis line 123 - Themes cannot really “emerge”; rather, they are constructed/produced by authors/researchers. See Ahmed et al., 2025:** [**https://www.sciencedirect.com/science/article/pii/S2949916X25000222**](https://url.uk.m.mimecastprotect.com/s/efVUCoy14uErM8NFViGFp7gaN?domain=sciencedirect.com) **Please adapt the language accordingly.**

We agree that themes cannot passively ‘emerge’ from the data but are constructed through the analytical process undertaken by the research team.

We have edited this to:

*“This analysis was initially based on the themes identified…”*

**16. Minor - Synthesis line 123 - “This analysis was initially based on the themes emerging from the five papers listed as ‘key papers’ “: I am unsure what authors refer to when writing “themes emerging”: themes generated by the authors of the included original papers? Of themes generated by the authors of this scoping review?**

See reply to 14.

**Additionally, “five papers listed as key papers” requires additional information: please explain what those key papers are (e.g., refer to the Results section to indicate that these papers will be presented later)**

This has been edited to

*“This analysis was initially based on the themes identified from papers whose primary focus was our research topic, and was then extended to the remaining papers (many of which only included some incidental data on our research topic).”*

**17. Major - Synthesis line 131 - Line 122 says “themes” (categories) were inductively divided into sub-themes. However, the aim of the study was to “to identify disruptions, adaptations” (lines 69-70)… which end up being the “themes.” This indicates that the analysis was not inductive but entirely deductive (the categories are the same as the predetermined goal). This would be acceptable if presented as a deductive content or thematic analysis rather than an inductive thematic analysis. Please address this as this is a major methodological error.**

Thank you for highlighting this important methodological distinction. Our intention was not to conduct a fully deductive analysis, rather the review questions defined the broad domain of interest (e.g. disruptions and adaptations in service delivery, lines 69-70) while analytical categories and themes were developed inductively from the included studies.

As described in the methods section (page 7), we followed the approach by Thomas and Harden (2008), where descriptive themes were developed through inductive coding of study findings, followed by analytical themes that moved beyond the content of individual studies to generate new interpretations. As such themes were constructed through the analytical process, rather than pre-determined. The line 122 saying "themes" refers to these inductively-derived descriptive categories, not pre-determined constructs.

We have a description of our analysis on page 7:

*“This thematic synthesis broadly followed Thomas and Harden’s [17] approach, adapted to the synthesis of secondary constructs (e.g. the authors’ interpretations of barriers/facilitators to service access) from both quantitative and qualitative data. Thomas and Harden’s approach includes inductive coding of findings from papers in order to develop ‘descriptive’ themes, and following this the development of ‘analytical’ themes where the reviewers go beyond the individual studies to develop new constructs or explanations that address the review questions.”*

This reflects our two-stage process: inductive theme generation followed by analytical synthesis within the broad domain defined by the review aims.

**18. Minor - Synthesis line 127 - Please explain briefly the Thomas & Harden approach and how it was used in this work.**

We have added more detail here (page 7):

“*Thomas and Harden’s approach includes coding findings from papers in order to develop ‘descriptive’ themes, and following this the development of ‘analytical’ themes where the reviewers go beyond the individual studies to develop new constructs or explanations.”*

**19. Major - Synthesis 134-135 - Repeating myself from the first round of comment, but this is key: “ii. women’s experiences seeking help with informal sources” is not a theme, it is a category. I highly recommend adapting the analysis section to represent seemed to have been done (content analysis rather than thematic analysis) OR working on the categories so they adequately represent themes (a theme, here, would clearly express what the “women’s experiences” are to represent the essence of those experiences, instead of having a broad term like “women’s experiences” that could mean literally anything [a category]).
This was a comment that I previously made in the first round, but is crucial, as what is presented as “thematic analysis” in this manuscript is not.**

We thank you for your feedback and have now edited the heading mentioned (page 29) to:

*“2.b.Turning to community and family networks for support”.*

Regarding the analysis section, as outlined above under our answer to 14, we have followed Thomas and Harden’s approach to thematic synthesis and this is now described on Page 7. Thomas and Harden explicitly describe descriptive themes as broad organisers of inductively coded findings from included studies, serving as "line-by-line coding containers" before analytical interpretation. This reflects our use of thematic synthesis to collate and interpret the evidence across studies, consistent with the approach outlined by Thomas and Harden (2008).

#### In the current manuscript, the major headings (e.g. “1. Health service delivery for VAW during COVID-19 (supply themes)”) therefore represent these descriptive thematic groupings, while sub-headings captures specific patterns observed across studies (sub-themes). The revised version (e.g. "Turning to community networks") now clearly expresses the pattern of behaviour rather than broad descriptors, consistent with thematic synthesis conventions.

**20. Major - Synthesis line 137 (and throughout the Results section) - As I now understand that authors analyzed secondary constructs only, I would strongly advise not using primary construct (direct participants’ quotes) to “illustrate authors findings”. If authors want to illustrate their findings (stemming from secondary constructs), I recommend using quotes from secondary constructs only, as this is what was analyzed. I think another reviewer recommended this as well.**

Thank you for this feedback. We have chosen to retain the verbatim participants quotes from the included studies because they are clearly attributed to their source studies (with references), and provide essential context for the secondary constructs that form our thematic synthesis.

As mentioned in the method section (page 7) this is a thematic synthesis of *secondary* constructs:

*“This thematic synthesis broadly followed Thomas and Harden’s [17] approach, adapted to the synthesis of secondary constructs (e.g. the authors’ interpretations of barriers/facilitators to service access) from both quantitative and qualitative data.”*

And that participant quotes are only included to illustrate these findings. This approach is consistent with Thomas and Harden (2008), who endorses using illustrative quotes from primary studies to enhance transparency when synthesising authors' interpretations.

**21. Minor - Results line 208- Thank you for this addition. Would it be possible to add the total number of studies where 1st author are from LMIC institutions?**

As some authors may be consultants at HIC institutions but based in an LMIC setting we have decided not to be specific about this level of detail.

**22. Minor - Results lines 215-217- I suggest adding the Arksey and O’Malley reference to support this claim.**

We assume you are referring to lines 115-117 under screening, we have added the Arksey and O’Malley reference here.

**23. Minor - Table 2, Study aim column - I suggest removing the “To” in front of all listed objectives/aim to lighten the table just a little bit.**

We prefer not to do this, for clarity of expression.

**24. Minor - Table 2 - The articles aren’t presented in alphabetical or chronological order in Table 2. I would advise using the alphabetical order.**

Table 2 has been reordered in alphabetical order.

**25. Minor - Throughout Results section - Please add the reference from which the quotes are from (that should be from secondary constructs only) at the end of the quote.**

References have now been added.

**26. Minor - Throughout Results section - Thank you for including for contextual information for the findings. When/if possible, please be more specific about the context of the findings as simply stating the country might not be enough (contexts can vary a lot inside the countries). For example, line 34 (young women participant, Kenya), should read (city of Kisumu, Kenya). Same applies with all quotes (e.g., “Before coronavirus I used to tell […]”; “There are families […]”) This would help better contextualize findings. Although I recommend removing quotes from primary constructs, this applies to quotes from secondary constructs if some were to be added.**

All quotes now mention the geographic location of the participant. The town has been edited in the quote mentioned (Kisumu, Kenya) and in all quotes where it is possible to do so. In some quotes this information is not available in the original paper and hence only the country has been mentioned.

**27. Minor - Results line 60 - Although well known, please define USAID acronym.**

This has been defined at first use on page 13.

**28. Minor - Results line 63 - The sentence “A USAID funded project […] average of 642%” is unclear (increased compared to what? A % cannot be a monthly count). Please rephrase (e.g., 642% average monthly increase in the identification, management, and response to GBV survivors, compared to pre-intervention levels)**

This has been edited to:

*“A USAID funded project in Kenya focussed on locally led solutions was reported by authors to have improved the identification, management, and response for gender-based violence survivors by a monthly average of 642% over a 10 month period [33].”*

**29. Minor - Results 2a (line 172)- I feel like the 2a “theme” phrasing is a bit clunky. Here are some suggestions: Evolving patterns of help-seeking; Variability in survivors’ engagement with services; Outbreak-driven shifts in survivors’ access to services**

#### This has been edited to:

#### 2.a. Changes in the number of survivors presenting at health services

**30. Minor - Discussion lines 298-299 - Please cite evidence showing that high-income countries got public/media attention earlier than LMICs for the information in parenthesis (“initially in high income[…]”).**

This has now been added.

**31. Minor Limitation section- You might want to add that the text screening process was done with a single reviewer (rather than two independent reviewers), which may have introduced a bias.**

We agree that single‑reviewer screening can introduce bias; however, in our case this process was already mitigated and transparently described in the Methods section.

“*Double screening by two co-authors (MC and JS) took place selectively at two different stages of the review, specifically for papers where there was some uncertainty over inclusion. At the title and abstract stage, two reviewers (MC and JS) double-screened 32 papers; and 4 papers at the full text screening stage.”*
Given this existing quality check, we do not consider single‑reviewer screening to be a significant limitation in this context.

**32. Minor - Discussion line 333 - Last sentence of paragraph doesn’t seem idiomatic (“linked with a review”) and is unclear to me. I recommend rephrasing.**

This has been edited to: “Despite significant focus on these two topics, they have been examined in parallel rather than together : therefore this scoping review provides a timely contribution.”

**33. Minor - Conclusions line 382 - Please specify what the ‘good practices’ refer to (e.g., VAW services good practices?)**

This has been edited to: “a broader range of health service delivery good practices.”

# Reviewer 3

**34. Reviewer #3: This second version of the article seems to me to be an improvement. We felt that the choice of low- and middle-income sites was not justified and constituted a bias in the study. Improvements have been made in this regard. I note an effort to improve the work. However, I remain convinced that the scientific contribution of this work would have been significant if the authors had opted for a comparative study between low-, medium-, and high-income situations. I believe that highlighting the different specificities of each country would have been of definite scientific interest and would be of paramount importance in terms of programming. In view of the efforts made, I think this article should be accepted with minor revisions.**

Thank you for this feedback. While we agree that in future reviews triangulation across contexts could indeed offer valuable insights. We want to reiterate that including evidence from high-income settings, where health systems are generally more resilient, would have obscured the context-specific barriers and responses that are most relevant to LMICs. LMIC settings was the focus of our review.

**Article report**

**Background**

**35. Lines: 62, 63, 64**

**For me, the scientific contribution of this study would have been a comparative analysis of the three types of environment. In my opinion, limiting the study to low and middle incomes does not seem justified, particularly given that the crisis was global. However, this could be a focus for your future research.**

We agree that it is an important area for future research, please see our reply above under 34.

**36. Lines 62 to 64.The authors still need to make an effort to clearly convey the objective of the research. With reference to what is proposed, I see two main objectives linked by 'and to'.**

We have edited this to:

*Our study aimed i. to understand how health services for violence against women (VAW) were affected in low- and middle-income (LMIC) settings during recent outbreaks, and ii. to understand women’s help-seeking for violence during these periods.*

**37. Lines 70 to 74: I think there are more than two specific questions. Check the wording.**

We have edited the wording:

*To address this objective we asked two sub-questions: i) to what extent health services for VAW were delivered during recent outbreaks, and what challenges and facilitators shaped service provision (supply side); ii) how women sought help for VAW during outbreaks and which sources of support they tried to access (demand side).*

**Search results**

**Characteristics of studies**

**38. In the first version of the paper, I recommended focusing exclusively on COVID-19. The authors have provided explanations, but I think you need to reconsider lines 174-175. If your study addresses a gap, whether scientific or empirical, this gap cannot be relevant if you are dealing with Ebola or Zika, as there is no data available. For a study covering three epidemics, there is only data related to one epidemic: COVID-19. If you cannot remove the other two cases (Ebola and Zika) from your work, please make an effort to show the methodological implications that this entails. What methodological lesson should be learned from this? In science, I think it is much more important to understand the reasons behind the choices made.**

Thank you for highlighting this important methodological consideration. An important methodological insight gained from our scoping review is the complete absence of published literature on VAW service delivery during non- COVID outbreaks (Ebola, Zika). This finding itself constitutes a substantial contribution , as we highlighted in the discussion (lines 326):*“There is a significant research gap in experiences from the range of other (non-COVID-19) outbreaks that have also shattered heath systems and exacerbated violence. This gap is striking considering that outbreak responses to Ebola (in West Africa and Democratic Republic of the Congo) and Zika (in the Americas) have also been critiqued as gender blind and as having neglected other essential services for women [54–57].”*

Including these outbreaks in our search strategy ensured comprehensive mapping of the available evidence base, per scoping review methodology (Arksey & O'Malley, 2005).

The ‘characteristics of studies’ section (page 156) transparently reports: *“All thirty two articles included focussed on experiences during the COVID-19 pandemic, no papers were found examining experiences during other outbreaks such as Zika or Ebola..”*

We believe that this approach reveals both what exists (COVID-19 data) and what is absent (non-COVID gaps), providing a complete evidence map rather than a COVID-only search that might have missed this critical gap – strengthening rather than weakening our paper’s contribution.

**39. Line 18- Despite the authors' explanations regarding the use of verbatim quotes, I believe that this method is inappropriate. I therefore reiterate my observation that the authors should remove the verbatim quotes. I reserve judgment when you include verbatim quotes in your work, which is exclusively a review of the literature. I believe that it is the results that are of interest. You can therefore do without verbatim quotes, which are not a result in themselves.**

Thank you for this feedback. We have chosen to retain the verbatim participants quotes from the included studies because they are clearly attributed to their source studies (with references), and provide essential context for the secondary constructs that form our thematic synthesis.

As mentioned in the method section (page 7) this is a thematic synthesis of *secondary* constructs:

*“This thematic synthesis broadly followed Thomas and Harden’s [17] approach, adapted to the synthesis of secondary constructs (e.g. the authors’ interpretations of barriers/facilitators to service access) from both quantitative and qualitative data.”*

And that participant quotes are only included to illustrate these findings. This approach is consistent with Thomas and Harden (2008), who endorses using illustrative quotes from primary studies to enhance transparency when synthesising authors' interpretations.

**40. Line 27- The concept of social distancing needs to be deconstructed. It is true that it has been used in public health to determine health measures, but opt for the concept of physical distancing for greater precision and empathy.**

This has been edited throughout (lines 36, 52, 25, 236, 371, 342).

**Discussion**

**41. Line 293, I think it would be appropriate to use the names of the epidemics: Ebola, Zika, COVID-19, instead of the word “recent epidemics.” It is impossible to systematically date the word “recent.” Your work must stand the test of time.**

We have edited this to:

“*Although our search focussed on multiple outbreaks (Ebola, Zika, COVID-19) over the 10 years prior to the search.”*

**General comments**

**This second version of the article seems to me to be an improvement. We felt that the choice of low- and middle-income sites was not justified and constituted a bias in the study. Improvements have been made in this regard. I note an effort to improve the work. However, I remain convinced that the scientific contribution of this work would have been significant if the authors had opted for a comparative study between low-, medium-, and high-income situations. I believe that highlighting the different specificities of each country would have been of definite scientific interest and would be of paramount importance in terms of programming. In view of the efforts made, I think this article should be accepted with minor revisions.**

Thank you for your comments.
